# Ginsenoside Rh2 Attenuates Intervertebral Disc Degeneration by Modulating the PI3K/AKT Signaling Pathway to Suppress Apoptosis

**DOI:** 10.3390/ijms27188228

**Published:** 2026-09-16

**Authors:** Bo Li, Qizhu Chen, Yichuan Chen, Huoliang Zheng, Linyu Jin, Leisheng Jiang, Xingfeng Zheng, Shengdan Jiang

**Affiliations:** Department of Spine Surgery, Xinhua Hospital, Shanghai Jiao Tong University School of Medicine, Shanghai 200092, China; libo01@xinhuamed.com.cn (B.L.); chenqz333@163.com (Q.C.); yichuan_chen0208@163.com (Y.C.); 13127829136@163.com (H.Z.); msofcn@126.com (L.J.); jiangleisheng@xinhuamed.com.cn (L.J.); zhengxinfeng@xinhuamed.com.cn (X.Z.)

**Keywords:** ginsenoside Rh2, intervertebral disc degeneration, nucleus pulposus cells, PI3K/AKT signaling pathway, extracellular matrix homeostasis, apoptosis

## Abstract

Intervertebral disc degeneration (IVDD) is a major cause of chronic low back pain and imposes a substantial socioeconomic burden. Ginsenoside Rh2 (Gin-Rh2), a bioactive ginseng compound with antioxidant properties, may have therapeutic potential in IVDD, but its effects and underlying mechanisms remain unclear. Here, we evaluated Gin-Rh2 in cellular and rat models of IVDD. In vitro, Gin-Rh2 attenuated interleukin-1β (IL-1β)-induced nucleus pulposus cell apoptosis, reduced matrix metalloproteinase-3 (MMP3) expression, and increased aggrecan and type II collagen production. Network pharmacology and molecular docking implicated the phosphoinositide 3-kinase/protein kinase B (PI3K/AKT) pathway as a potential target. Mechanistic experiments further indicated that inhibition of PI3K/AKT signaling contributed to the protective effects of Gin-Rh2, whereas pharmacological activation of this pathway partially attenuated these effects. In vivo, Gin-Rh2 attenuated puncture-induced disc degeneration. Collectively, these findings suggest that Gin-Rh2 protects nucleus pulposus cells and attenuates experimental IVDD, supporting its further investigation as a potential therapeutic candidate.

## 1. Introduction

Intervertebral disc degeneration (IVDD) is a leading cause of low back pain and disability, driven by progressive structural deterioration and impaired disc function [1,2]. With an aging population, its socioeconomic burden continues to escalate [3]. Current therapies remain largely symptomatic, and no disease-modifying treatment exists [4]. Therefore, developing new therapeutic strategies that can attenuate disc degeneration and promote intervertebral disc repair has become a current research priority.

The intervertebral disc is composed of the superior and inferior cartilaginous endplates, the peripheral annulus fibrosus (AF), and the central nucleus pulposus (NP) [5]. Disruption of nucleus pulposus cell (NPC) homeostasis is a hallmark of IVDD, leading to persistent oxidative stress, extracellular matrix (ECM) degradation, and apoptosis [6]. The ECM surrounding NPCs is composed predominantly of collagens (primarily type I and type II collagen), elastin, proteoglycans (particularly aggrecan), and glycoproteins such as fibronectin and laminin [7]. During IVDD, the composition and structural organization of the ECM undergo profound remodeling, characterized by excessive collagen degradation, aggrecan depletion, and reduced water content [8,9]. These changes further exacerbate oxidative stress and cell apoptosis, establishing a vicious cycle that ultimately culminates in loss of disc function and the development of associated clinical symptoms [10]. Research has shown that during the progression of IVDD, excessive generation of reactive oxygen species (ROS) and the resulting oxidative stress are direct causes of apoptosis in NPCs and also constitute a key driver of accelerated ECM degradation [11]. Therefore, identifying a pharmacological agent capable of reducing oxidative stress and suppressing apoptosis is of critical importance for stabilizing the intervertebral disc microenvironment.

Given the central role of oxidative stress in IVDD, increasing attention has been directed toward natural bioactive monomers and natural product–derived pharmacology, which may modulate redox imbalance and downstream cell death pathways. Traditional Chinese medicine (TCM) represents a valuable source of natural products and bioactive compounds for contemporary pharmacological research [12,13]. Several TCM-derived interventions, including Lycium barbarum, Yiqi Huoxue Recipe, and (Z)-ligustilide, have shown protective effects in experimental models of inflammation-associated IVDD [14,15,16]. These studies highlight the potential of TCM constituents in enhancing therapeutic efficacy for IVDD. Historically, ginseng has attracted considerable attention due to its broad therapeutic effects. Among these, ginsenoside Rh2 (Gin-Rh2), as an active component of ginseng, exhibits potent anti-inflammatory and antioxidant activities [17,18], and has been investigated in the treatment of cancer [19], airway inflammation [20], and cardioprotection [21]. Moreover, Gin-Rh2 may exert antioxidant effects through the FOXO3a–KEAP1–NRF2 axis [22], Nrf2/HO-1 axis [23], and TGF-β1/Smad axis [24]. More recently, Xuan et al. reported that Gin-Rh2 protected NPCs and reduced the severity of experimental IVDD-related changes by promoting HIF-1α-mediated autophagy, suppressing pyroptosis, and preserving ECM homeostasis [25]. However, the mechanisms by which Gin-Rh2 attenuates oxidative stress and apoptosis in NPCs during IVDD remain incompletely understood. Accordingly, the present study investigated the effects of Gin-Rh2 on oxidative stress, apoptosis, and ECM metabolism in NPCs and explored the underlying molecular mechanisms.

**Scheme 1:**
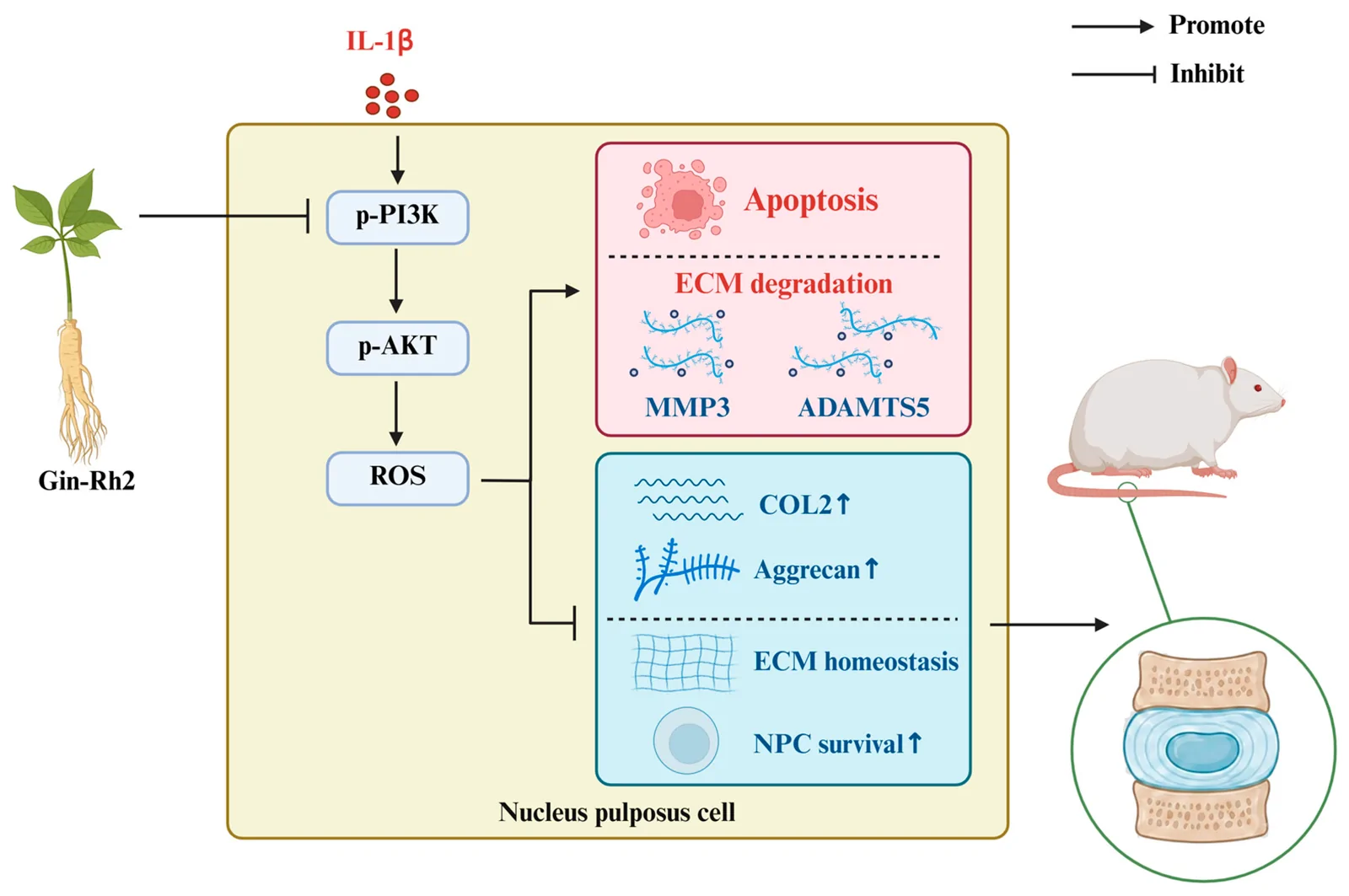
Gin-Rh2 attenuates IVDD by suppressing PI3K/AKT-mediated oxidative stress in nucleus pulposus cells.

Here, we integrated network pharmacology and bioinformatic analyses with in vitro and in vivo experiments to investigate the therapeutic potential of Gin-Rh2 in IVDD (Scheme 1). These analyses identified PI3K/AKT signaling as a candidate pathway, which was subsequently examined through molecular docking and cellular experiments. Our findings indicate that Gin-Rh2 attenuates disc degeneration by reducing NPC apoptosis and ECM degradation. Mechanistic experiments further implicate suppression of PI3K/AKT signaling in the cellular protective effects of Gin-Rh2. Together, these findings support Gin-Rh2 as a promising candidate for IVDD treatment.

## 2. Results

### 2.1. Screening and Identification of Potential Targets of Gin-Rh2

**Figure 1:**
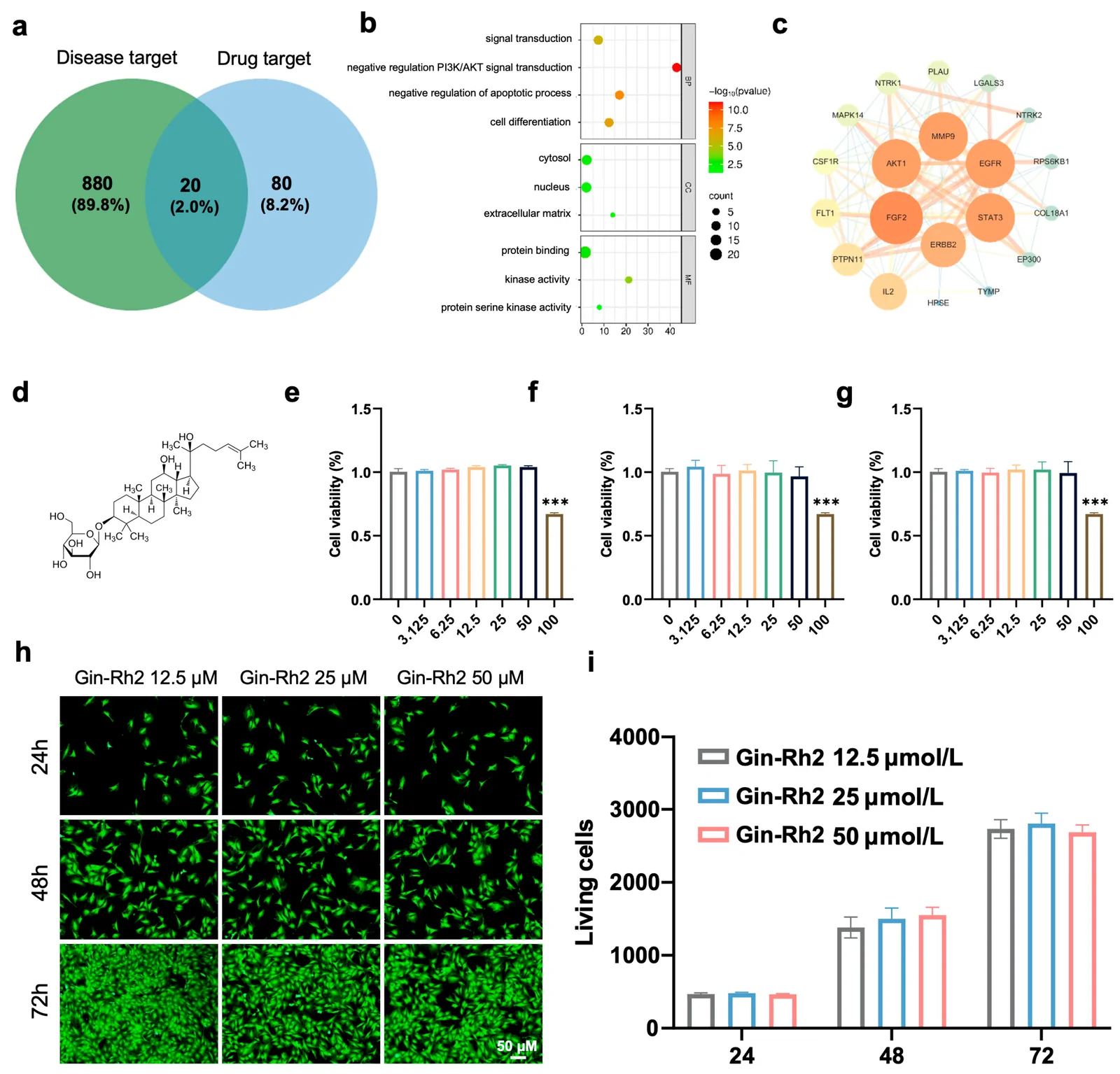
Network pharmacology identifies potential Gin-Rh2 targets and assesses in vitro biocompatibility. (**a**) Venn diagram showing the overlap between the potential targets of Gin-Rh2 and the disease targets of IVDD. (**b**) GO enrichment analysis for the common targets. (**c**) Visualization of the PPI network of the common targets. (**d**) The chemical structure of Gin-Rh2. (**e**–**g**) CCK-8 assay was used to evaluate the effects of Gin-Rh2 on NP cell viability (n = 3). (**h**) Live/dead staining was performed to observe cell growth. Representative fluorescence images of cells treated with Gin-Rh2 at 12.5, 25, and 50 μM for 24 h, 48 h, and 72 h are shown (n = 3). (**i**) Quantification of the number of live cells in different treatment groups at 24, 48, and 72 h. *** *p* < 0.001.

To identify the potential targets of Gin-Rh2 in the treatment of IVDD, we first used the SwissTargetPrediction database to predict 100 potential targets of Gin-Rh2. Meanwhile, using the GeneCards database, we collected 900 disease-related targets associated with IVDD. Cross-analysis between the potential targets of Gin-Rh2 and the IVDD disease targets (Figure 1a) revealed 20 common targets. These overlapping targets were considered candidate mediators of the therapeutic effects of Gin-Rh2 in IVDD.

To further investigate the biological functions and pathways of these 20 common targets, we performed Gene Ontology (GO) enrichment analysis. The Biological Process enrichment results indicated that these targets were significantly enriched in signal transduction, the negative regulation of PI3K/AKT signal transduction, the negative regulation of apoptotic process, and cell differentiation. The Cellular Component analysis suggested that these targets were mainly located in the cytosol, nucleus, and ECM. The Molecular Function analysis showed that these targets were primarily involved in protein binding, kinase activity, and protein serine kinase activity (Figure 1b). Overall, these data suggested the possible involvement of processes such as signal transduction, apoptosis, differentiation, and ECM metabolism in NPCs in the therapeutic effects of Gin-Rh2 on IVDD.

To further explore the interaction relationships among these 20 common targets and identify key nodes, we constructed a protein–protein interaction (PPI) network (Figure 1c). PPI network analysis showed that AKT served as a core target in this network and closely interacted with multiple other targets. In combination with the GO enrichment results, the PI3K/AKT signaling pathway is a classic anti-oxidative pathway that plays an important role in cellular protection, anti-oxidative stress, and the treatment of various diseases. Based on these findings, we hypothesized that the potential therapeutic effect of Gin-Rh2 on IVDD may be achieved by targeting the PI3K/AKT signaling pathway, thereby reducing oxidative stress, inhibiting apoptosis, and helping restore normal ECM metabolism in NPCs.

### 2.2. In Vitro Biocompatibility and Cytotoxicity of Gin-Rh2 in NPCs

To better investigate the therapeutic efficacy of Gin-Rh2, we first determined its safe dose range and safe exposure time in NPCs. We assessed the biocompatibility of Gin-Rh2 using Cell Counting Kit-8 (CCK-8) (Figure 1e–g). We found that at 24, 48, and 72 h, only when the concentration reached 100 μM did the viability of NPCs significantly decrease; the other concentrations showed no obvious effects on cell viability. In addition, live/dead staining fluorescence showed that treatment with 50 μM did not affect the normal proliferation and viability of NPCs (Figure 1h,i). Therefore, we selected 50 μM for subsequent experiments.

### 2.3. Gin-Rh2 Restores ECM Homeostasis in IL-1β(Interleukin-1 Beta)-Treated NPCs

**Figure 2:**
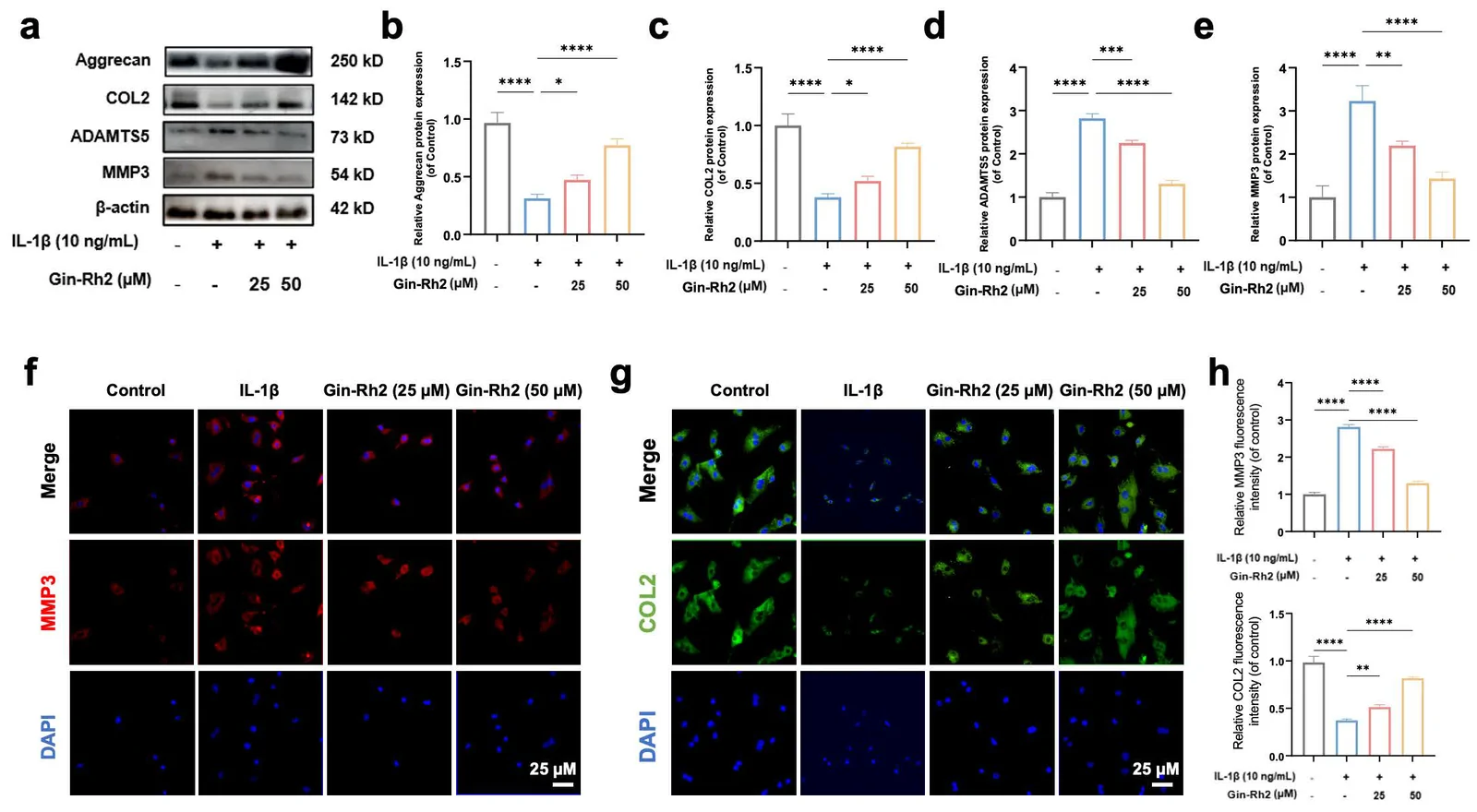
Gin-Rh2 suppresses IL-1β–induced ECM degradation–associated marker changes in NPCs. (**a**) Western blot analysis of aggrecan, COL2, ADAMTS5, and MMP3 (n = 3). (**b**–**e**) Semi-quantitative analysis of the expression of aggrecan, COL2, ADAMTS5, and MMP3. (**f**) Immunofluorescence staining for MMP3 and DAPI (n = 3). (**g**) Immunofluorescence staining for COL2 and DAPI (n = 3). (**h**) Quantification of fluorescence intensity of MMP3 and COL2. * *p* < 0.05, ** *p* < 0.01, *** *p* < 0.001, and **** *p* < 0.0001.

To assess the therapeutic potential of Gin-Rh2 for IVDD, NPCs were stimulated with IL-1β (10 ng/mL) to mimic the inflammatory microenvironment of the degenerative NP and subsequently treated with different concentrations of Gin-Rh2 [26]. Key proteins involved in ECM anabolism and catabolism were then evaluated by western blotting. The results showed that, following treatment with IL-1β, the NPCs increased expression of ECM-degrading proteins, including a disintegrin and metalloproteinase with thrombospondin motifs 5 (ADAMTS5) and matrix metalloproteinase 3 (MMP3). In contrast, the expression of matrix-synthesis–related proteins, such as aggrecan and Type II collagen (COL2), was significantly downregulated, indicating that ECM metabolism was shifted toward degradation. Notably, upon the addition of Gin-Rh2, these alterations were attenuated; aggrecan and COL2 expression increased, while ADAMTS5 and MMP3 expression decreased. Moreover, with increasing concentrations of Gin-Rh2, these effects became more pronounced, suggesting that Gin-Rh2 promotes a metabolic shift of ECM toward synthesis (Figure 2a–e). Immunofluorescence further corroborated these findings, demonstrating that Gin-Rh2 mitigated ECM degradation induced by oxidative stress (Figure 2f–h).

### 2.4. Gin-Rh2 Dose-Dependently Inhibits IL-1β-Induced Apoptosis in NPCs

**Figure 3:**
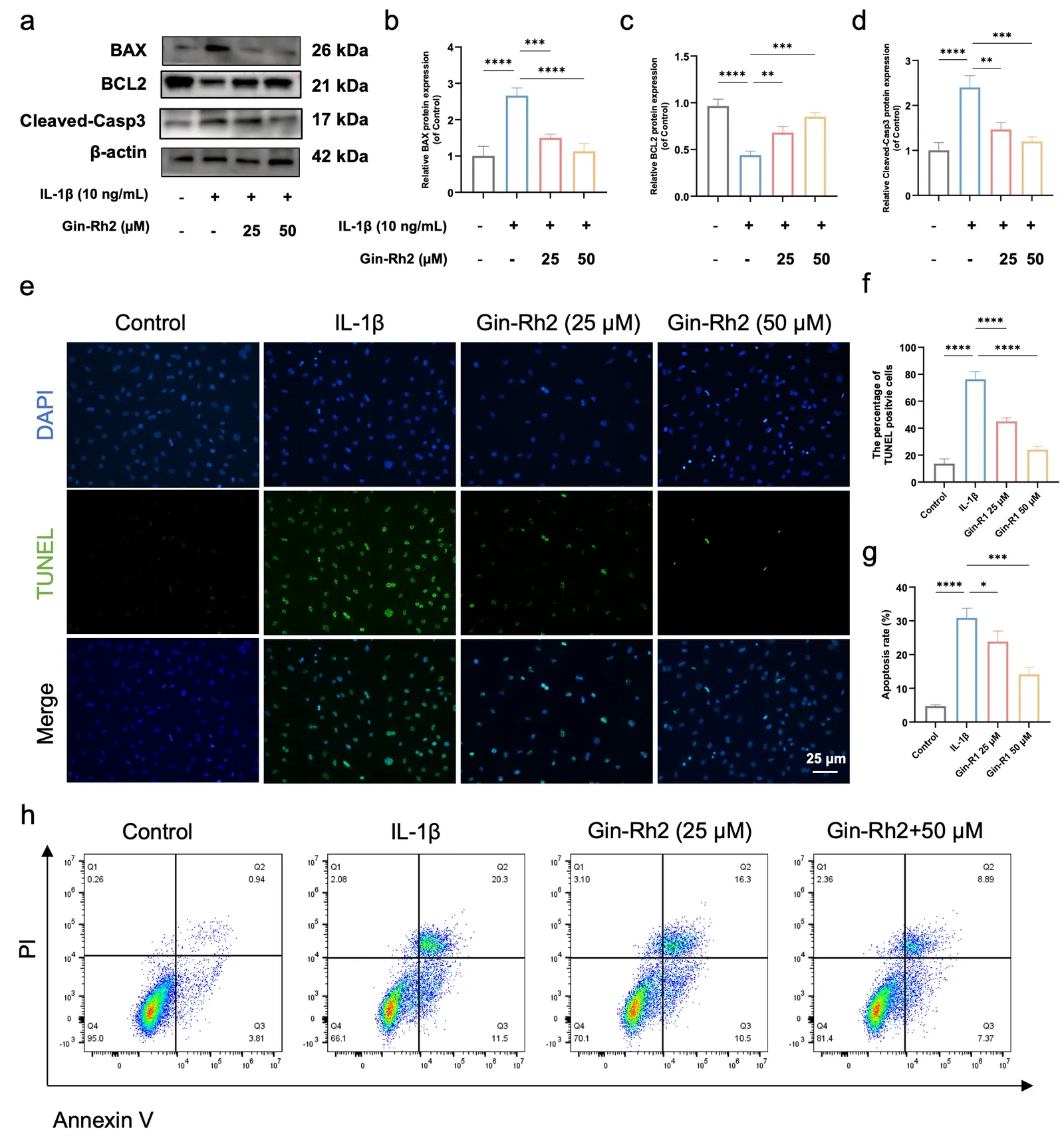
Gin-Rh2 suppresses IL-1β-induced apoptosis in NPCs. (**a**) Western blot analysis of BAX, BCL2, and Cleaved-Casp3 (n = 3). (**b**–**d**) Relative protein expression of BAX, BCL2, and Cleaved-Casp3. (**e**) TUNEL staining for apoptosis (n = 3). (**f**) Quantification of the percentage of TUNEL-positive cells and apoptosis-related results. (**g**,**h**) Quantitative analysis of apoptotic cells and flow cytometry analysis of apoptosis phenotypes using Annexin V/PI (n = 3). * *p* < 0.05, ** *p* < 0.01, *** *p* < 0.001, and **** *p* < 0.0001.

As suggested by the target gene enrichment analysis in Figure 1, Gin-Rh2 exhibits anti-apoptotic potential. To verify this hypothesis, NPCs were treated with IL-1β to induce apoptosis, followed by intervention with different doses of Gin-Rh2. Western blot analyses revealed that, upon IL-1β stimulation, the apoptosis-associated proteins BAX (BCL2-associated X protein) and Cleaved caspase-3 (Cleaved-Casp3) were significantly upregulated, while the anti-apoptotic protein B-cell lymphoma 2 (BCL2) was downregulated. Concurrently, Gin-Rh2 treatment dose-dependently suppressed the IL-1β–induced increases in BAX and Cleaved-Casp3, and dose-dependently increased BCL2 expression (Figure 3a–d). These results suggest that Gin-Rh2 may exert a protective effect on NPCs by modulating apoptosis-related signaling pathways.

Furthermore, after co-treatment with IL-1β and Gin-Rh2, apoptosis was evaluated at both the level of DNA damage and the proportion of apoptotic cells using Terminal deoxynucleotidyl transferase dUTP nick-end labeling (TUNEL) staining and flow cytometry. The TUNEL results demonstrated that the apoptotic cell rate was markedly increased after IL-1β treatment, whereas the number of TUNEL-positive cells significantly decreased following Gin-Rh2 administration (Figure 3e,f). In agreement with the TUNEL findings, flow cytometry further confirmed that Gin-Rh2 inhibited the apoptotic process. Specifically, IL-1β significantly increased the proportions of early apoptotic cells (Annexin V-FITC (Fluorescein isothiocyanate) +/PI−, Q3) and late apoptotic/necrotic cells (Annexin V-FITC+/PI+, Q2). After Gin-Rh2 treatment, both the early apoptotic (Q3) and late apoptotic/necrotic (Q2) fractions were significantly reduced compared with the IL-1β group (Figure 3g,h).

### 2.5. Molecular Docking of Gin-Rh2 with PI3K

**Figure 4:**
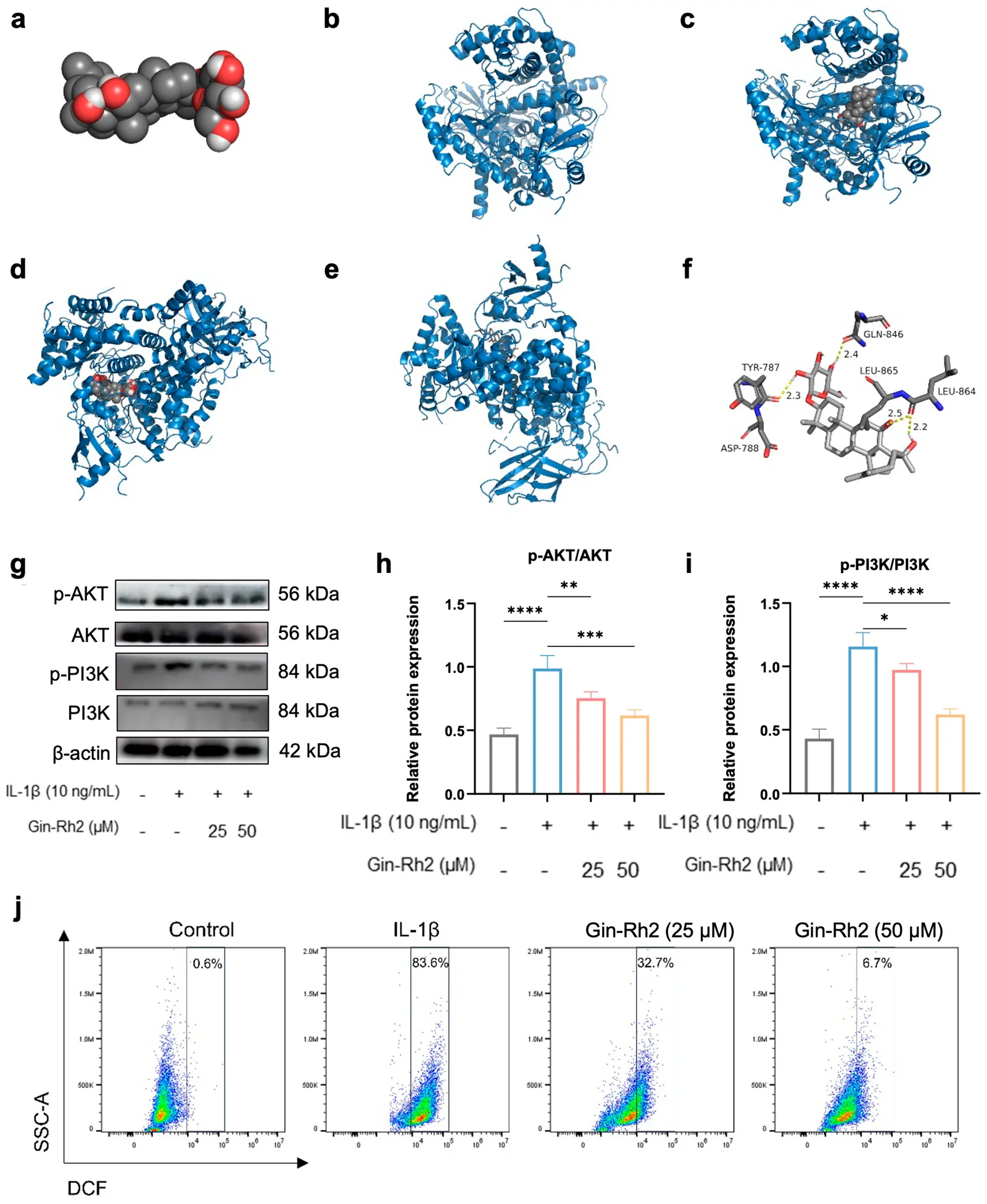
Molecular docking of Gin-Rh2 with PI3K and its regulatory effects on IL-1β-induced PI3K/AKT pathway activation and ROS levels. (**a**) Schematic illustration of the 3D structure of Gin-Rh2. (**b**–**e**) Molecular docking poses of Gin-Rh2 with PI3K (from different perspectives) are shown to indicate that Gin-Rh2 can enter a putative PI3K binding pocket and form stable interactions. (**f**) Schematic diagram of the interactions between Gin-Rh2 and key binding sites of PI3K. (**g**) Western blot and (**h**,**i**) semi-quantitative analysis of PI3K/AKT proteins (n = 3). (**j**) Flow cytometry analysis of intracellular ROS levels (n = 3). * *p* < 0.05, ** *p* < 0.01, *** *p* < 0.001, and **** *p* < 0.0001.

Based on the network pharmacology analysis, PI3K was identified as a potential molecular target of Gin-Rh2. Molecular docking was subsequently performed to explore the potential interaction and predicted binding mode between Gin-Rh2 and PI3K. The molecular structure of Gin-Rh2 is shown in Figure 4a. The α-helical and β-sheet domains of PI3K are presented from a top-view perspective in Figure 4b. The predicted docking pose of Gin-Rh2 within PI3K is illustrated in Figure 4c–e.

The docking analysis generated a predicted pose in which Gin-Rh2 occupied a putative binding pocket of PI3K. In this computational model, Gin-Rh2 was predicted to interact with several amino acid residues, including Tyr787, Asp788, GLN-846, Leu864, and Leu865. These in silico findings suggest a potential interaction between Gin-Rh2 and PI3K but do not demonstrate direct physical binding. Specifically, Gin-Rh2 forms hydrogen bonds with TYR-787 and ASP-788, with bond lengths of approximately 2.3 Å. In addition, Gin-Rh2 forms a hydrogen bond with GLN-846 (approximately 2.4 Å). Moreover, hydrophobic contacts are observed between Gin-Rh2 and LEU-864 and LEU-865, with the two residues positioned approximately 2.2 Å and 2.5 Å from the ligand, respectively (Figure 4f).

These relatively short hydrogen-bond distances are compatible with a predicted binding pose for Gin-Rh2 in the PI3K pocket. Hydrophobic residues such as LEU-864 and LEU-865 may further contribute to the ligand–protein association, although docking alone does not quantify binding stability or specificity.

### 2.6. Inhibition of the PI3K/AKT Signaling Pathway by Gin-Rh2

Based on our previous bioinformatic analyses, we hypothesized that Gin-Rh2 may inhibit the PI3K/AKT signaling pathway. Molecular docking results further supported this hypothesis, showing a predicted binding pose between Gin-Rh2 and PI3K. To further examine whether Gin-Rh2 modulates PI3K/AKT signaling, we treated NPCs with different concentrations of Gin-Rh2 and assessed the phosphorylation levels of PI3K and AKT via western blot. Compared with the control group, IL-1β significantly increased the ratios of p-AKT/AKT and p-PI3K/PI3K, indicating that IL-1β potently activates the PI3K/AKT signaling pathway. Following the addition of 25 μM and 50 μM Gin-Rh2 under IL-1β stimulation, both ratios were reduced, with statistically significant differences and a dose-dependent trend (Figure 4g–i). Given the important role of the PI3K/AKT signaling pathway in regulating ROS, and that ROS are key factors inducing apoptosis in NPCs, we further examined intracellular ROS levels using flow cytometry. The results showed that IL-1β stimulation significantly increased intracellular ROS levels, whereas Gin-Rh2 dose-dependently reduced IL-1β-induced ROS production (Figure 4j). These findings demonstrate that Gin-Rh2 inhibits IL-1β-induced activation of the PI3K/AKT signaling pathway and reduces intracellular ROS levels.

### 2.7. Gin-Rh2 Exerts Anti-Apoptotic Effects via PI3K/AKT Signaling

**Figure 5:**
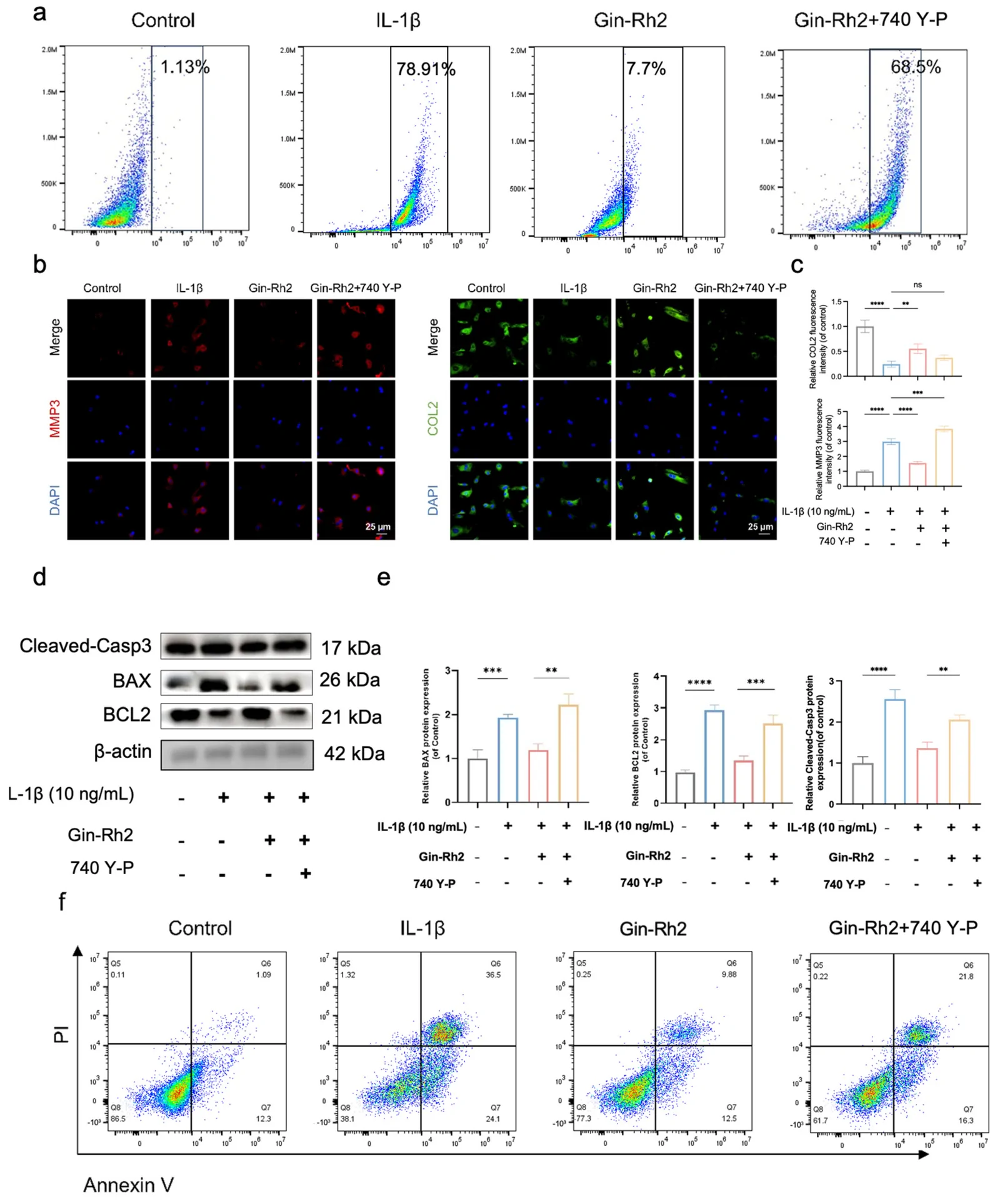
The 740 Y-P withdraws the protective effects of Gin-Rh2 on IL-1β–induced apoptosis and ECM degradation in NPCs. (**a**) Flow cytometry analysis of intracellular ROS levels (n = 3). (**b**,**c**) Immunofluorescence images (**b**) and quantification intensity (**c**) of MMP3 and COL2 (n = 3). (**d**,**e**) Western blot and semi-quantitative analysis of BAX, BCL2 and Cleaved-Casp3 (n = 3). (**f**) Flow cytometry analysis of Annexin V/PI (n = 3). ** *p* < 0.01, *** *p* < 0.001, and **** *p* < 0.0001; ns, not significant (*p* ≥ 0.05).

We further employed a PI3K activator (740 Y-P, 20 μM [27]) to determine whether PI3K signaling is responsible for the anti-apoptotic effect of Gin-Rh2 in NPCs. First, intracellular ROS levels were quantified by flow cytometry. Compared with the Gin-Rh2 group, the 740 Y-P-treated condition exhibited a marked increase in ROS, indicating that PI3K activation largely impaired the ROS-scavenging capacity of Gin-Rh2 (Figure 5a). In line with this, PI3K activation abolished the protective effect of Gin-Rh2 against IL-1β-induced ECM degradation, as revealed by MMP3 and COL2 immunofluorescence (Figure 5b,c). Consistently, western blot analysis showed that PI3K activation caused increases in BAX and Cleaved-Casp3, accompanied by a decrease in BCL2 (Figure 5d,e). Moreover, Annexin V/PI flow cytometry confirmed that IL-1β significantly promoted early and late apoptosis, whereas Gin-Rh2 substantially reduced IL-1β-triggered cell death; importantly, the 740 Y-P withdrew these effects (Figure 5f). Overall, these data indicate that Gin-Rh2 suppresses IL-1β-induced apoptosis through PI3K/AKT-dependent signaling, and activation of PI3K functionally counteracts Gin-Rh2-mediated cytoprotection.

### 2.8. Gin-Rh2 Ameliorates IVDD in a Rat Model

**Figure 6:**
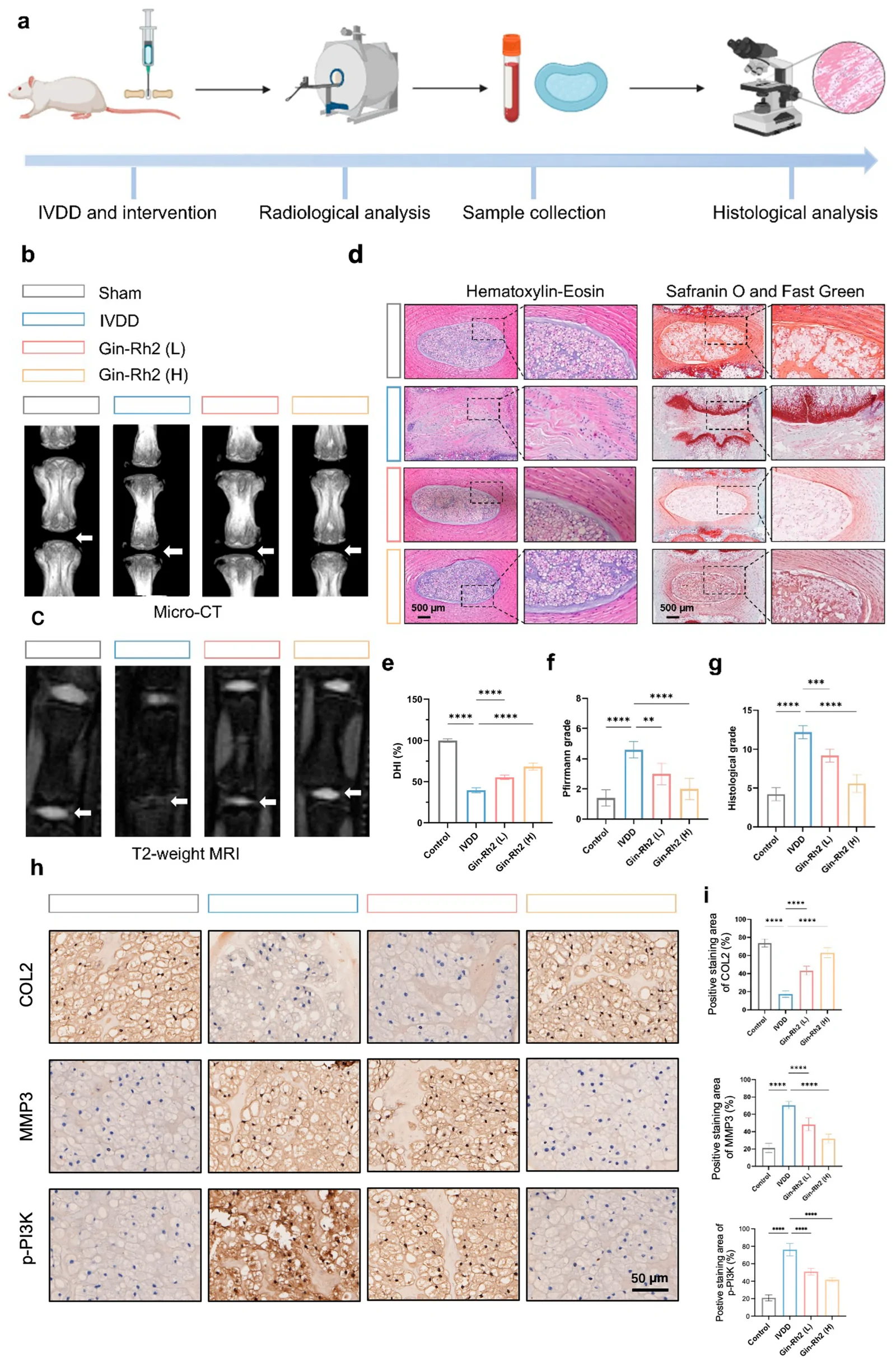
Gin-Rh2 alleviates puncture-induced IVDD by modulating radiological findings, histological phenotypes, and related molecular expression. (**a**) Schematic diagram of rat puncture model. (**b**) Representative micro-CT images from different groups (Sham, IVDD, Gin-Rh2 (L), and Gin-Rh2 (H)), the white arrows indicated the treated intervertebral discs. (n = 6). (**c**) T2-weighted MRI images from different groups (n = 6). (**d**) H&E staining and SO/FG staining were used to evaluate NPC morphology and matrix/cartilage-like component deposition (n = 6). (**e**–**g**) Quantitative analysis of DHI, Pfirrmann grade and histology. (**h**) Representative images of immunohistochemistry staining for COL2, MMP3 and p-PI3K (n = 6). (**i**) Quantitative analysis of immunohistochemistry staining intensity. ** *p* < 0.01, *** *p* < 0.001, and **** *p* < 0.0001.

To establish an in vivo IVDD model, we punctured the intervertebral discs of rats to induce intradiscal degenerative changes. Thereafter, rats were administered Gin-Rh2 at low (Gin-Rh2 (L), 2 mg/kg) and high (Gin-Rh2 (H), 8 mg/kg) doses by daily intraperitoneal injection, while the sham and IVDD groups received an equivalent volume of normal saline. At the end of week 8, the intervertebral discs were harvested for imaging and histological analysis to evaluate the therapeutic effects of Gin-Rh2 in vivo (Figure 6a).

IVDD is characterized by micro-computed tomography (micro-CT) decreased intervertebral space height and loss of water content within the disc. Under imaging, compared with the sham group, the IVDD group showed a marked reduction in the disc height index (DHI). After Gin-Rh2 treatment, the DHI was improved, and the height increased in a dose-dependent manner with higher Gin-Rh2 doses (Figure 6b).

Magnetic resonance imaging (MRI) is considered the gold standard for IVDD diagnosis. In healthy IVDs, the hydrated NP tissue appears as high signal intensity on T2-weighted images. During disc degeneration, ECM remodeling results in reduced water content and volume, and in cases of severe degeneration, MRI often displays a “black disc”. MRI analysis showed that, compared with the sham group, the IVDD group exhibited loss of disc signal and a prominent black disc. In contrast, the Gin-Rh2 (L) group and the Gin-Rh2 (H) group showed signal retention within the disc (Figure 6c).

We further performed hematoxylin and eosin (H&E) staining to evaluate the cellular structures and morphology of the NP, AF and endplate (Figure 6d). In the IVDD group, we observed a substantial reduction in NPC content. The NP region was replaced by fibrous cells, the AF displayed disordered lamellar organization, and cartilage endplate fractures were more frequent. With Gin-Rh2 treatment, the number of NPCs showed only slight changes; the boundary between the NP and AF remained clear, and there were no obvious alterations in the AF or the cartilage endplate. Next, Safranin O/Fast Green (SO/FG) staining was used to assess proteoglycan/cartilage matrix in the IVD. Proteoglycans appeared as red-brown, whereas collagen appeared blue-green. Compared with the sham group, the IVDD group exhibited a significant reduction in red staining in the NP tissue (Figure 6d). With increasing doses of Gin-Rh2, proteoglycan content within the disc was significantly improved (Figure 6e–g).

To further demonstrate that Gin-Rh2 promotes ECM synthesis and prevents degradation, immunohistochemistry analyses were performed to determine the expression levels of COL2 and MMP3 in NP tissue. The results showed that, compared with the sham group, the IVDD group exhibited a significant increase in MMP3-positive staining area and a marked decrease in COL2-positive staining area. Compared with the IVDD group, Gin-Rh2 treatment dose-dependently increased COL2-positive staining and decreased MMP3-positive staining (Figure 6h,i). Furthermore, in order to examine whether Gin-Rh2 is associated with PI3K-related signaling in vivo, we performed immunohistochemical staining for p-PI3K and found that Gin-Rh2 can reduce the over-activated PI3K in the degeneration process.

## 3. Discussion

IVDD is a critical pathological mechanism underlying chronic low back pain and poses a major public health challenge worldwide in terms of disability and healthcare resource consumption [28,29]. Although IVDD can be treated with conservative therapies and symptom-guided surgical interventions, these strategies mainly alleviate pain and cannot attenuate IVDD or restore spinal mechanical function [30]. Here, we show that Gin-Rh2, a diol-type low–sugar chain saponin monomer derived from ginseng, effectively attenuates ECM degradation, oxidative stress and NPC apoptosis, thereby stabilizing the NP cellular microenvironment and ultimately slowing the progression of IVDD. Mechanistic studies based on molecular pharmacology suggest that the potential therapeutic target of Gin-Rh2 in IVDD involves inhibition of the PI3K/AKT signaling pathway. Molecular docking predicted a plausible binding pose between Gin-Rh2 and PI3K, and PI3K-activator experiments further support the involvement of the PI3K/AKT axis for the protective effects mediated by Gin-Rh2. In addition, using a needle puncture-induced rat IVDD model, we demonstrate that Gin-Rh2 treatment produces significant in vivo therapeutic efficacy. Collectively, our findings indicate that Gin-Rh2 represents a preclinical candidate therapeutic agent for alleviating and potentially attenuating IVDD progression.

For more than two millennia, administered TCMs have been widely used to treat low back pain, primarily following therapeutic strategies of tonifying the kidney, dispelling “wind-cold,” eliminating dampness, and resolving blood stasis [31,32]. With advances in chemistry, chemical biology, and molecular biology, several naturally derived plant products have been reported to prevent ECM dysfunction and to attenuate the progression of bone and joint degenerative diseases in cellular and animal models [33]. Existing studies have shown that trionochinene E can promote lysosomal biogenesis and enhance autophagy by activating transcription factor EB and transcription factor E3. This process helps protect NPCs from oxidative stress and improves IVDD [34]. Yiqi Huoxue Fang, a widely used prescription formula, can promote the formation of the Beclin1–VPS34 complex. By activating upstream AMPK and upregulating the deubiquitinase USP13, it triggers autophagy and thereby alleviates the occurrence and development of IVDD [15].

Gin-Rh2, one of the active constituents of ginseng, has been reported to play an important role in the treatment of many diseases. Prior studies indicate that Gin-Rh2 is frequently used in therapeutic regimens for inflammatory diseases. For example, in mouse BV-2 microglial cells, Gin-Rh2 inhibits lipopolysaccharide/interferon-γ–induced NO production, as well as the expression of iNOS, COX-2, TNF-α, and IL-1β through modulation of the protein kinase A/AP-1 signaling pathway [35]. In addition, Gin-Rh2 suppresses TNF-α–induced ICAM-1 expression by inhibiting the activity of NF-κB and the JNK/AP-1 signaling pathways in human astrocytes [36]. More recently, Xuan et al. investigated Gin-Rh2 from the perspective of hypoxia-related signaling. They reported that Gin-Rh2 promoted HIF-1α-mediated autophagy, thereby reducing NPC pyroptosis and ECM damage during IVDD [25]. Although this study provided evidence for the protective effects of Gin-Rh2 in IVDD, the HIF-1α–autophagy–pyroptosis axis may represent only one component of its biological actions. IVDD is a multifactorial process involving oxidative stress, NPC apoptosis, and disrupted ECM homeostasis. Whether Gin-Rh2 also regulates these pathological processes, and which signaling pathways mediate these effects, remained incompletely understood. Therefore, further investigation was warranted to obtain a more comprehensive understanding of the mechanisms underlying the effects of Gin-Rh2 in IVDD.

In this study, we initially performed network pharmacology analyses to predict the potential targets of Gin-Rh2 for the treatment of IVDD. Using the GeneCards database, we conducted an intersection analysis between the predicted targets of Gin-Rh2 and the therapeutic targets associated with IVDD. The results revealed 20 common targets. We then performed GO enrichment analyses on these targets. The findings indicated that the candidate therapeutic genes of Gin-Rh2 were significantly enriched in pathways related to signal transduction, the PI3K/AKT signaling pathway, the process of apoptosis, and cell differentiation. These results suggest that Gin-Rh2 may exert therapeutic effects on IVDD through multiple targets and multiple pathways. Based on these results, we further verified in vitro whether Gin-Rh2 can improve NPC viability and thereby attenuate IVDD. First, we demonstrated that Gin-Rh2 did not show significant cytotoxicity in NPCs treated with 50 μM for a prolonged period, as assessed by CCK-8 and live/dead staining.

To investigate whether Gin-Rh2 can treat IVDD, we used IL-1β to mimic the microenvironment of IVDD. We evaluated NPC activity by detecting proteins related to ECM synthesis and ECM degradation. The results showed that, upon stimulation with IL-1β, NPCs secreted high levels of ECM degradation-associated proteins, including MMP3 and ADAMTS5, while the levels of ECM synthesis-associated proteins COL2 and aggrecan were markedly decreased. These findings indicate that NPCs exhibit an ECM degradation tendency in the degenerative microenvironment of IVDD. Notably, after treatment with Gin-Rh2 at different doses, this degradation tendency was attenuated, and NPCs shifted toward an ECM synthesis direction. These results indicate that Gin-Rh2 produces a significant improvement in NPC viability. Excessive apoptosis of NPCs is a key pathological event in IVDD, contributing to ECM degradation and progressive loss of disc function. In the present study, we investigated whether Gin-Rh2 could protect NPCs against IL-1β-induced apoptosis. Western blot analysis demonstrated that Gin-Rh2 markedly reduced the expression of the pro-apoptotic proteins BAX and Cleaved-Casp3 while increasing the expression of the anti-apoptotic protein BCL2. These findings were further supported by flow cytometric analysis and TUNEL staining, both of which confirmed that Gin-Rh2 significantly attenuated IL-1β-induced NPC apoptosis in a dose-dependent manner. Given that excessive apoptosis disrupts ECM homeostasis and accelerates disc degeneration, the anti-apoptotic effect of Gin-Rh2 may contribute substantially to its protective role in IVDD.

To further investigate the potential antioxidant mechanism of Gin-Rh2 in IVDD, we performed network pharmacology analysis combined with GeneCards, together with GO enrichment. The predicted action targets of Gin-Rh2 were significantly enriched in PI3K-related pathways, suggesting a possible involvement of the PI3K/AKT signaling axis. Previous studies have shown that Gin-Rh2 can suppress lung pathological changes, lung edema, inflammatory cell infiltration, and the release of multiple pro-inflammatory cytokines by inhibiting PI3K/AKT, thereby attenuating acute lung injury [20]. This indicates that Gin-Rh2 may prevent the onset of IVDD by modulating PI3K/AKT. The PI3K/AKT pathway is a core intracellular signal transduction cascade. Signals such as growth factors or insulin activate PI3K on the cell membrane, which catalyzes the generation of the second messenger PIP3 [37]. PIP3 then recruits and activates AKT [38]. Activated AKT further phosphorylates multiple downstream targets, thereby regulating cell proliferation, survival, metabolism, and angiogenesis [39]. Although basal or transient PI3K/AKT activation generally promotes cell survival, its effects appear to depend on the pathological context and downstream signaling branches. Under certain inflammatory conditions, aberrant or sustained PI3K/AKT activation may facilitate NF-κB and NLRP3 inflammasome signaling, thereby amplifying inflammatory mediator production, extracellular matrix catabolism and inflammatory cell death [40,41]. Dysregulation of the PI3K/AKT pathway plays an important role in the development of IVDD. Accordingly, modulation of PI3K/AKT signaling has been investigated as a potential context-dependent strategy for IVDD treatment [42]. For example, maslinic acid alleviated IVDD by inhibiting the PI3K/AKT pathway and downregulating MMPs and ADAMTS, thereby alleviating ECM imbalance [43]. Maltol has also been reported to inhibit ECM degradation and inflammatory responses by suppressing PI3K/AKT, increasing the expression of anabolic proteins while decreasing catabolic proteins, and reducing secretion of inflammatory mediators such as IL-18 and IL-1β, thereby attenuating IVDD [44].

To verify whether Gin-Rh2 potentially regulates PI3K, the docking results showed that Gin-Rh2 fit into the PI3K binding pocket, forming hydrogen bonds with TYR-787, ASP-788, and GLN-846 (bond lengths: 2.3 Å, 2.3 Å, and 2.4 Å) and making hydrophobic contacts with LEU-864 and LEU-865 (contact distances: ~2.2 Å and ~2.5 Å), respectively. These relatively short interaction distances are consistent with the predicted binding pose of Gin-Rh2 within the PI3K binding pocket. This suggests that Gin-Rh2 may modulate the PI3K/AKT pathway. In our cellular experiments, IL-1β stimulation significantly increased the levels of p-PI3K/PI3K and p-AKT/AKT compared with the control group. Gin-Rh2 treatment reduced this activation in a concentration-dependent manner, demonstrating that Gin-Rh2 inhibits the PI3K/AKT pathway in NPCs under oxidative stress conditions. Functionally, Gin-Rh2 also attenuated intracellular ROS accumulation in a concentration-dependent manner. To confirm whether this pathway is required for the protective effects of Gin-Rh2, we used a 740 Y-P. Western blot, flow cytometry, and immunofluorescence showed that PI3K activation largely counteracted the anti-apoptotic effect of Gin-Rh2, increased ROS levels, and promoted ECM degradation. Collectively, these integrated computational and experimental findings indicate that Gin-Rh2 attenuates IVDD progression, with modulation of PI3K/AKT signaling contributing to its protective effects.

The innovation of this study is that we apply Gin-Rh2 to the treatment of IVDD. By integrating network pharmacology prediction with molecular docking, we identified PI3K as a candidate target and provided experimental evidence for the involvement of PI3K/AKT signaling through which Gin-Rh2 exerts its effects. We further highlighted intervertebral disc microenvironment remodeling by examining the effects of Gin-Rh2 on ECM, oxidative stress, and apoptosis in NPCs. In addition, we systematically demonstrated that, within the IVDD microenvironment, Gin-Rh2 may inhibit the progression of IVDD by suppressing the PI3K/AKT pathway, providing a new direction for the development of therapeutic drugs. However, this study still has limitations. Although molecular docking suggested a potential interaction between Gin-Rh2 and PI3K, direct binding was not experimentally validated in this study. Further biochemical, cellular, and structural studies are required to determine whether Gin-Rh2 physically interacts with PI3K, identify any potential binding sites and associated conformational changes, and establish whether Gin-Rh2 affects the association of PI3K with regulatory subunits such as p85. We have shown that Gin-Rh2 suppresses apoptosis in NPCs via the PI3K/AKT pathway, but downstream regulation may also involve other processes such as autophagy; whether Gin-Rh2 synergistically exerts its effects by inducing protective autophagy remains to be elucidated. Moreover, the in vivo doses of 2 and 8 mg/kg were selected from a previously reported intraperitoneal dose range in rats [45], rather than by conversion from the 50 μM concentration used in vitro. Because plasma exposure and intradiscal Gin-Rh2 concentrations were not measured, whether these regimens achieved intradiscal concentrations comparable to 50 μM remains unknown. Given the relatively low bioavailability of Gin-Rh2 [46], future studies should characterize its pharmacokinetics and disc-tissue distribution. Local sustained-release delivery systems or structural modifications should also be explored to improve its concentration and retention within the intervertebral disc.

## 4. Materials and Methods

### 4.1. Bioinformatic Prediction

The chemical structure of Gin-Rh2 (PubChem CID: 119307) was downloaded from the PubChem database. Its potential protein targets were predicted using the SwissTargetPrediction web server with the species set to Homo sapiens and a minimum probability threshold of 0.1. Disease-related targets for IVDD were retrieved from the GeneCards database using the query “intervertebral disc degeneration”. Only entries with a relevance score ≥ 10 were retained. After merging the two target lists and removing duplicates, the official gene symbols were standardized using the HGNC database. The intersection targets were then visualized with Cytoscape v3.10.0, and a PPI network was constructed using STRING v12.0 (Homo sapiens, confidence cutoff 0.40, hiding disconnected nodes). GO enrichment analysis was performed in R version 4.4.1 using the clusterProfiler package (v4.10.0) with the org.Hs.eg.db background set. *p*-values were adjusted by the Benjamini–Hochberg method, and only terms with an adjusted *p* < 0.05 were considered significant.

### 4.2. Molecular Docking

Molecular docking between Gin-Rh2 and PI3K was carried out using the CB-Dock2 web server. The protein structure of PI3K (PDB ID: 1E7U) was obtained from the RCSB PDB. The Gin-Rh2 ligand (PubChem CID: 119307) was prepared by energy minimization. Docking was run with a semi-flexible protocol where the PI3K protein was kept rigid while all rotatable bonds of the ligand were allowed to rotate freely. The binding box was centered on the predicted cavity. The resulting docking poses were ranked by the Vina score. The best-ranked pose was visualized using PyMOL (v3.1.0, Schrödinger, LLC). Hydrogen bond distances and the amino acid residues forming the binding pocket were identified and measured.

### 4.3. Cell Isolation and Culture

Rat NP cells were isolated from male Sprague–Dawley rats (6-week-old, 200–250 g). Briefly, rat intervertebral disc fragments were minced and digested with 0.1% type II collagenase in DMEM/F12 for 4 h at 37 °C. After digestion, the tissue pieces were seeded in complete medium (DMEM/F12 supplemented with 10% fetal bovine serum (Gibco, NY, USA), 100 U/mL penicillin and 100 μg/mL streptomycin (Gibco, NY, USA). Cells were maintained at 37 °C in a humidified 5% CO_2_ atmosphere.

### 4.4. Cell Viability Assay

Cell viability was evaluated using a CCK-8 (Beyotime, Shanghai, China). Briefly, NPCs were seeded in 96-well plates at a density of 5 × 10^3^ cells/well. After attachment, Gin-Rh2 (MeRCK, Darmstadt, Germany) was dissolved in dimethyl sulfoxide (DMSO) to prepare a stock solution and subsequently diluted with culture medium to final concentrations of 3.125, 6.25, 12.5, 25, 50, or 100 μM. The cells were treated with the indicated concentrations of Gin-Rh2 for 24, 48, or 72 h. The final concentration of DMSO was maintained at the same level in all experimental groups. At the end of treatment, the medium was replaced with 100 μL fresh DMEM/F12 containing 5 μL CCK-8 solution. Following incubation for 1 h at 37 °C, the absorbance was recorded at 450 nm using a microplate reader (Multiskan SkyHigh, Thermo Fisher, Waltham, MA, USA).

### 4.5. Western Blot

NPCs were seeded in six-well plates and allowed to adhere. The cells were then treated as follows: the control group received an equivalent volume of vehicle (PBS containing the same final concentration of DMSO as the treatment groups). The IL-1β group received IL-1β together with the same vehicle, whereas the IL-1β + Gin-Rh2 groups were treated with IL-1β followed by different concentrations of Gin-Rh2. For the 740 Y-P intervention group, cells were pretreated with 20 μM 740 Y-P (Solarbio, China) for 2 h, subsequently exposed to 10 ng/mL IL-1β, and then treated with Gin-Rh2. The final concentration of DMSO was maintained at the same level in all experimental groups. After 24 h of treatment, the cells were harvested. Cells were lysed in RIPA buffer (Beyotime, China) containing 1 mM phenylmethylsulfonyl fluoride (PMSF). Lysates were centrifuged at 12,000× *g* for 15 min at 4 °C, and the supernatant protein concentration was determined with a BCA protein assay kit (Beyotime, China). Equal amounts of protein (20–30 μg per lane) were separated by 8–12% SDS-PAGE (Vazyme, Nanjing, China) and transferred to polyvinylidene difluoride (PVDF) membranes. Membranes were blocked with 5% non-fat milk in TBST (Tris-buffered saline containing 0.1% Tween-20) for 1.5 h at room temperature and then incubated overnight at 4 °C with the following primary antibodies. After washing three times with TBST, membranes were incubated with horseradish peroxidase (HRP)-conjugated goat anti-rabbit IgG (Beyotime, China) or goat anti-mouse IgG (Beyotime, China) for 2 h at room temperature. After a further three washes, immunoreactive bands were visualized using an enhanced chemiluminescence (ECL) substrate (Beyotime, China) and captured on a ChemiDoc imaging system (Bio-Rad, Hercules, CA, USA). Band intensities were quantified with ImageJ software 1.8.0. The relative protein expression was normalized to that of β-actin. The following primary antibodies were adopted: COL2 (ab34712, 1:1000), ADAMTS5 (ab41037, 1:1000) and BAX (ab32503, 1:1000), which were obtained from Abcam (Cambridge, UK). MMP3 (17873-1-AP, 1:1000) Aggrecan (13880-1-AP, 1:1000), Cleaved-Casp3 (25128-1-AP, 1:1000), BCL2 (26593–1-AP, 1:1000), PI3K (20662-1-AP, 1:1000) and β-actin (66009–1-Ig, 1:3000) were purchased from Proteintech Group (Wuhan, China). The p-PI3K antibody (bs-10277R, 1:1000) was purchased from Bioss (Beijing, China). AKT (AF6261, 1:1000) and p-AKT (AF0016, 1:1000) were purchased from Affinity (Nanjing, China).

### 4.6. TUNEL Fluorescent Staining

Apoptotic cells were detected using a TUNEL Apoptosis Assay Kit (Beyotime, China), according to the manufacturer’s protocol. NPCs were grown on coverslips in 6-well plates. A total of 50 μL of TUNEL detection reagent was added to the cells, followed by incubation at 37 °C in the dark for 60 min. The samples were then washed three times with PBS, mounted with anti-fade fluorescence mounting medium, and observed under a fluorescence microscope (Carl Zeiss Inc., Zeiss Axiovert A1, White Plains, NY, USA).

### 4.7. Flow Cytometry

Cell apoptosis was also assessed by flow cytometry using an Annexin V-FITC/PI Apoptosis Detection Kit (Vazyme, China). After the designated treatments, both detached and adherent cells were collected, washed with cold PBS, and resuspended in 195 μL of 1× binding buffer. Annexin V-FITC (5 μL) and propidium iodide (10 μL) were added, and the mixture was incubated for 20 min at room temperature in the dark. Stained cells were immediately analyzed on a BD FACSCanto II flow cytometer (BD Biosciences, San Jose, CA, USA). Data were processed using FlowJo software (v10.8).

### 4.8. ROS Measurement

Intracellular ROS levels were measured using a ROS Assay Kit (Beyotime, China). In brief, after treatment, NPCs were washed twice with PBS and loaded with 10 μM 2′,7′-dichlorodihydrofluorescein diacetate in serum-free DMEM/F12 for 20 min at 37 °C. Cells were then washed three times with serum-free medium to remove excess probe. For quantitative analysis, cells were trypsinised, resuspended in PBS, and subjected to flow cytometry on the BD FACSCanto II instrument.

### 4.9. Immunofluorescence Staining

Cells grown on coverslips were fixed, permeabilized with 0.1% Triton X-100 for 15 min as above and blocked with 5% bovine serum albumin (BSA) for 1 h. They were then incubated overnight at 4 °C with primary antibodies (COL2 (Abcam, ab34712, 1:200) or MMP3 (Abcam, ab52915, 1:200)). After washing, coverslips were incubated for 1 h at room temperature with Alexa Fluor 488- or 594-conjugated secondary antibodies (Invitrogen, 1:500). Nuclei were labelled with DAPI. Images were captured using a confocal laser scanning microscope (Leica TCS SP8, Leica Microsystems, Wetzlar, Germany).

### 4.10. Live/Dead Staining

Cell viability was also visualized using a Calcein-AM/PI Double Staining Kit (Beyotime, C2015). Following the kit’s instructions, NPCs were incubated with 2 μM Calcein-AM and 4.5 μM PI for 15 min at 37 °C. Live cells (green fluorescence) and dead cells (red fluorescence) were imaged immediately with a fluorescence microscope.

### 4.11. Animal Experiments

All animal procedures were approved by the Experimental Animal Ethics Committee of Xinhua Hospital Affiliated with Shanghai Jiao Tong University (XHEC-F-2025-038) and are reported in accordance with the ARRIVE guidelines. Male Sprague–Dawley rats (6 weeks old, 200–250 g) were purchased from S Shanghai SLAC Laboratory Animal Co., Ltd. (Shanghai, China). After one week of acclimation, rats were randomly allocated into four groups (n = 6 per group): sham, IVDD, IVDD + low-dose Gin-Rh2 (2 mg/kg), and IVDD + high-dose Gin-Rh2 (8 mg/kg). Rats were housed in an SPF facility under controlled conditions (12 h light/dark cycle, 22–26 °C, 40–70% relative humidity) with free access to standard rodent chow and tap water.

All surgical procedures were performed under aseptic conditions. Rats were anesthetised with 2% pentobarbital sodium (40 mg/kg, i.p.). A 27 G needle was inserted perpendicularly through the AF to a depth of 4 mm and kept in place for 1 min to induce disc degeneration. During the post-operative period, animals were monitored at appropriate intervals for general condition, body weight, wound status, and signs of pain or distress. Analgesia was provided as part of the approved protocol, and humane endpoints were applied to minimize suffering. From post-operative day 3, rats in the Gin-Rh2 groups received daily intraperitoneal injections of Gin-Rh2 (2 mg/kg or 8 mg/kg) in normal saline [45]. Sham and IVDD groups received equal volumes of normal saline (5 mL/kg).

### 4.12. Micro-CT and MRI Assessments

Micro-CT scanning of the tail was performed using a Xradia 520 Versa system (Zeiss, Cambridge, UK) at 50 kV and 160 μA with an isotropic voxel size of 15 μm. Three-dimensional reconstructions were generated, and the DHI was calculated using the formula DHI (%) = (mean disc height/mean height of adjacent vertebral bodies) × 100%, as previously described [47]. Immediately after micro-CT, MRI was carried out on a 3.0 T clinical scanner (Achieva, Philips) using a dedicated small-animal coil. T2-weighted sagittal images were acquired (turbo spin echo, repetition time/echo time = 3000/85 ms, slice thickness 2 mm). Three orthopaedic researchers, blinded to group allocation, independently evaluated the images. The degree of disc degeneration was graded according to the Pfirrmann grading system, and the disc height was also scored.

### 4.13. Histological Evaluation

At post-operative week 8, rats were euthanized, and intervertebral disc tissues were collected. Specimens were fixed in 4% paraformaldehyde for 24 h, decalcified in 10% EDTA (pH 7.4) for 8 weeks at room temperature with constant shaking, and then embedded in paraffin. Serial mid-sagittal sections (5 μm) were cut to include the endplate, AF and NP.

For H&E staining, sections were deparaffinized and rehydrated, stained with hematoxylin for 5 min, differentiated with acid-alcohol, and then counterstained with eosin for 3 min. Afterward, sections were processed through routine dehydration and clearing, mounted, and observed under a microscope. For SO/FG staining, sections were deparaffinized and rehydrated and sequentially stained with Safranin O for 5 min followed by Fast Green for 5 min. Then sections were dehydrated, cleared, mounted, and examined histologically [47].

### 4.14. Immunohistochemistry

Immunohistochemical staining was performed on paraffin-embedded sections. Briefly, sections were deparaffinized and rehydrated, followed by antigen retrieval using pepsin. Endogenous peroxidase activity was quenched with hydrogen peroxide, and non-specific binding was blocked with serum. Sections were then incubated with the primary antibody (COL2, Abcam, ab34712, 1:200; MMP3, Abcam, ab52915, 1:200; p-PI3K, Bioss, bs-10277R, 1:200) overnight at 4 °C, followed by incubation with an appropriate HRP-conjugated secondary antibody (1:200). Immunoreactivity was visualized using DAB as the chromogen and counterstained with hematoxylin. After dehydration, clearing, and mounting, sections were examined and images were captured under a Digital pathology slide scanner (LG-FS80, Servicebio., Wuhan, China).

### 4.15. Statistical Analysis

Data are presented as the mean ± SD. Statistical analysis was performed using GraphPad Prism 9.0 and R version 4.4.1. Parametric data were compared by one-way or two-way ANOVA, followed by Tukey’s or Šídák’s post hoc tests as appropriate. For repeated measurements, two-way repeated measures ANOVA was applied. Pfirrmann grade scores were analyzed using the Kruskal–Wallis test, followed by Dunn’s multiple comparisons test. Statistical significance is indicated as follows: * *p* < 0.05, ** *p* < 0.01, *** *p* < 0.001, and **** *p* < 0.0001; ns, not significant (*p* ≥ 0.05).

## 5. Conclusions

In summary, our findings show that Gin-Rh2 attenuates NPC apoptosis, ECM degradation, and oxidative stress while reducing IL-1β-associated PI3K/AKT activation. Network pharmacology identified PI3K as a potential target, and molecular docking suggested a possible interaction between Gin-Rh2 and PI3K. Experimentally, Gin-Rh2 decreased PI3K and AKT phosphorylation, whereas pharmacological pathway activation partially attenuated its protective effects. These results suggest that modulation of PI3K/AKT signaling contributes, at least in part, to the protective activity of Gin-Rh2. In vivo, Gin-Rh2 attenuated IVDD-associated degenerative changes and helped preserve ECM homeostasis. These findings support further investigation of Gin-Rh2 as a potential therapeutic candidate and highlight the potential of TCM-derived bioactive compounds for IVDD treatment.

## Data Availability

The original contributions presented in this study are included in the article. Further inquiries can be directed to the corresponding author.

## Abbreviations

The following abbreviations are used in this manuscript:

ADAMTS5	A disintegrin and metalloproteinase with thrombospondin motifs 5
AF	Annulus fibrosus
AKT	Protein kinase B
AMPK	AMP-activated protein kinase
ANOVA	Analysis of variance
ARRIVE	Animal Research: Reporting of In Vivo Experiments
BAX	BCL2-associated X protein
BCA	Bicinchoninic acid
BCL-2	B-cell lymphoma 2
BSA	Bovine serum albumin
CCK-8	Cell Counting Kit-8
COL2	Type II collagen
DAB	3,3′-Diaminobenzidine
DAPI	4′,6-Diamidino-2-phenylindole
DHI	Disc height index
DMEM/F12	Dulbecco’s Modified Eagle Medium/Nutrient Mixture F-12
DMSO	Dimethyl sulfoxide
ECM	Extracellular matrix
ECL	Enhanced chemiluminescence
EDTA	Ethylenediaminetetraacetic acid
FBS	Fetal bovine serum
FITC	Fluorescein isothiocyanate
Gin-Rh2	Ginsenoside Rh2
GO	Gene Ontology
H&E	Hematoxylin and eosin
HRP	Horseradish peroxidase
IL-1β	Interleukin-1 beta
IVDD	Intervertebral disc degeneration
micro-CT	Micro-computed tomography
MMP3	Matrix metalloproteinase 3
MRI	Magnetic resonance imaging
NP	Nucleus pulposus
NPC	Nucleus pulposus cell
PBS	Phosphate-buffered saline
PI	Propidium iodide
PI3K	Phosphoinositide 3-kinase
PIP3	Phosphatidylinositol 3,4,5-trisphosphate
PKA	Protein kinase A
PMSF	Phenylmethylsulfonyl fluoride
PPI	Protein–protein interaction
PVDF	Polyvinylidene difluoride
RIPA	Radioimmunoprecipitation assay
ROS	Reactive oxygen species
SD	Standard deviation
SDS-PAGE	Sodium dodecyl sulfate–polyacrylamide gel electrophoresis
SO/FG	Safranin O/Fast Green
TBST	Tris-buffered saline containing Tween-20
TCM	Traditional Chinese medicine
TUNEL	Terminal deoxynucleotidyl transferase dUTP nick-end labeling

## References

[1] Knezevic N.N., Candido K.D., Vlaeyen J.W.S., Van Zundert J., Cohen S.P. (2021). Low back pain. Lancet.

[2] The Lancet Rheumatology (2023). The global epidemic of low back pain. Lancet Rheumatol..

[3] Hancock M., Kongsted A. (2024). Towards improving the Global Burden of Disease estimates for low back pain. Lancet Rheumatol..

[4] Samanta A., Lufkin T., Kraus P. (2023). Intervertebral disc degeneration-Current therapeutic options and challenges. Front. Public Health.

[5] Raj P.P. (2008). Intervertebral disc: Anatomy-physiology-pathophysiology-treatment. Pain Pract..

[6] He R., Wang Z., Cui M., Liu S., Wu W., Chen M., Wu Y., Qu Y., Lin H., Chen S. (2021). HIF1A Alleviates compression-induced apoptosis of nucleus pulposus derived stem cells via upregulating autophagy. Autophagy.

[7] Hammoor B.T., Lai C.S., Xiong G.X., Elliott D.M., Snyder B., Vresilovic E., Bono C.M., Freedman B.R. (2026). Intervertebral disc degeneration. Nat. Rev. Dis. Primers.

[8] Zhu Q., Gao X., Brown M.D., Temple H.T., Gu W. (2017). Simulation of water content distributions in degenerated human intervertebral discs. J. Orthop. Res..

[9] Ząbek Z., Wyczałkowska-Tomasik A., Poboży K., Adamus J.P., Turek G., Ząbek M., Pączek L. (2025). Understanding the Microenvironment of Intervertebral Disc Degeneration: A Comprehensive Review of Pathophysiological Insights and Therapeutic Implications. Int. J. Mol. Sci..

[10] Liang H.Z., Luo R.J., Li G.C., Zhang W.F., Song Y., Yang C. (2022). The Proteolysis of ECM in Intervertebral Disc Degeneration. Int. J. Mol. Sci..

[11] Chen X.L., Zhang A.R., Zhao K.C., Gao H.Y., Shi P.Z., Chen Y.H., Cheng Z.R., Zhou W.J., Zhang Y.K. (2024). The role of oxidative stress in intervertebral disc degeneration: Mechanisms and therapeutic implications. Ageing Res. Rev..

[12] Ma D., Wang S., Shi Y., Ni S., Tang M., Xu A. (2021). The development of traditional Chinese medicine. J. Tradit. Chin. Med. Sci..

[13] Marshall A. (2020). Traditional Chinese Medicine and Clinical Pharmacology. Drug Discovery and Evaluation: Methods in Clinical Pharmacology.

[14] Dou H.C., Chen Y.T., Chen J.X., Zhu Y.X., Yang S., Yan Y.Z., Lin Y., Hu S.L. (2025). Lycium barbarum alleviates oxidative stress-induced ferroptosis and enhances mitophagy in intervertebral disc degeneration. Cell. Signal..

[15] Dai F., Yu P.F., Yu Z.H., Jiang H., Ma Z.J., Liu J.T. (2021). Yiqi Huoxue Recipe Delayed Intervertebral Disc Degeneration by Activating Autophagy. Front. Pharmacol..

[16] Wang J.L., Fan C.Y., Zhang Y., Hua D., Ji Z.W., He W., Deng Y.K., Geng D.C., Wu X.X., Mao H.Q. (2025). (Z)-Ligustilide alleviates intervertebral disc degeneration by suppressing nucleus pulposus cell pyroptosis via Atg5/NLRP3 axis. Sci. China-Life Sci..

[17] Zhou G., Wang C.Z., Mohammadi S., Sawadogo W.R., Ma Q., Yuan C.S. (2023). Pharmacological Effects of Ginseng: Multiple Constituents and Multiple Actions on Humans. Am. J. Chin. Med..

[18] Attele A.S., Wu J.A., Yuan C.S. (1999). Ginseng pharmacology: Multiple constituents and multiple actions. Biochem. Pharmacol..

[19] Chen F., Deng Z., Xiong Z., Zhang B., Yang J., Hu J. (2015). A ROS-mediated lysosomal-mitochondrial pathway is induced by ginsenoside Rh2 in hepatoma HepG2 cells. Food Funct..

[20] Hsieh Y.H., Deng J.S., Chang Y.S., Huang G.J. (2018). Ginsenoside Rh2 Ameliorates Lipopolysaccharide-Induced Acute Lung Injury by Regulating the TLR4/PI3K/Akt/mTOR, Raf-1/MEK/ERK, and Keap1/Nrf2/HO-1 Signaling Pathways in Mice. Nutrients.

[21] Yu T., Xu J., Wang Q., Han X., Tu Y., Wang Y., Luo W., Wang M., Liang G. (2023). 20(S)-ginsenoside Rh2 inhibits angiotensin-2 mediated cardiac remodeling and inflammation associated with suppression of the JNK/AP-1 pathway. Biomed. Pharmacother..

[22] Wu J., Wang S., Zhao W., Li M., Li S. (2023). Ginsenoside Rh2 inhibits CBP/p300-mediated FOXO3a acetylation and epilepsy-induced oxidative damage via the FOXO3a-KEAP1-NRF2 pathway. Eur. J. Pharmacol..

[23] Zhu Y., Li J., Dai L., Feng W. (2025). Ginsenoside Rh2 Alleviate Sepsis-related Encephalopathy via Up-regulating Nrf2/HO-1 Pathway and Apoptosis Inhibition. Cell Biochem. Biophys..

[24] Vinoth Kumar R., Oh T.W., Park Y.K. (2016). Anti-Inflammatory Effects of Ginsenoside-Rh2 Inhibits LPS-Induced Activation of Microglia and Overproduction of Inflammatory Mediators Via Modulation of TGF-β1/Smad Pathway. Neurochem. Res..

[25] Xuan J., Chen J., Liu H., Chen Y., Zhang Z. (2026). Ginsenoside-Rh2 Alleviates Pyroptosis of Nucleus Pulposus Cells by Activating Autophagy via HIF-1α to Retard the Progression of Intervertebral Disc Degeneration. J. Cell. Physiol..

[26] Brand F.J., Forouzandeh M., Kaur H., Travascio F., de Rivero Vaccari J.P. (2016). Acidification changes affect the inflammasome in human nucleus pulposus cells. J. Inflamm..

[27] Liang H., Yang S., Huang Y., Zhu Y., Wu Q., Wu Z., Li S., Shi Y., Chen Z., Jin H. (2026). PTPN22 as a therapeutic target in intervertebral disc degeneration: Modulating mitophagy and pyroptosis through the PI3K/AKT/mTOR axis. J. Adv. Res..

[28] Onuora S. (2023). Low back pain is a growing concern. Nat. Rev. Rheumatol..

[29] (2023). GBD 2021 Low Back Pain Collaborators. Global, regional, and national burden of low back pain, 1990-2020, its attributable risk factors, and projections to 2050: A systematic analysis of the Global Burden of Disease Study 2021. Lancet Rheumatol..

[30] Xin J., Wang Y., Zheng Z., Wang S., Na S., Zhang S. (2022). Treatment of Intervertebral Disc Degeneration. Orthop. Surg..

[31] Zhu L., Yu C., Zhang X., Yu Z., Zhan F., Yu X., Wang S., He F., Han Y., Zhao H. (2020). The treatment of intervertebral disc degeneration using Traditional Chinese Medicine. J. Ethnopharmacol..

[32] Shim G.C., Lee S.-H., Lee Y.J., Lee S.W., Ha I.-H. (2025). Effects of traditional east asian herbal medicine decoctions on low back pain: A systematic review and meta-analysis. Eur. J. Integr. Med..

[33] Duan Y., Su Y.-T., Ren J., Zhou Q., Tang M., Li J., Li S.-X. (2023). Kidney tonifying traditional Chinese medicine: Potential implications for the prevention and treatment of osteoporosis. Front. Pharmacol..

[34] Niu Z., Tang G., Wang X., Yang X., Zhao Y., Wang Y., Liu Q., Zhang F., Zhao Y., Ding X. (2023). Trigonochinene E promotes lysosomal biogenesis and enhances autophagy via TFEB/TFE3 in human degenerative NP cells against oxidative stress. Phytomedicine.

[35] Bae E.-A., Kim E.-J., Park J.-S., Kim H.-S., Ryu J.H., Kim D.-H. (2006). Ginsenosides Rg3 and Rh2 inhibit the activation of AP-1 and protein kinase A pathway in lipopolysaccharide/interferon-gamma-stimulated BV-2 microglial cells. Planta Medica.

[36] Choi K., Kim M., Ryu J., Choi C. (2007). Ginsenosides compound K and Rh2 inhibit tumor necrosis factor-α-induced activation of the NF-κB and JNK pathways in human astroglial cells. Neurosci. Lett..

[37] Vanhaesebroeck B., Stephens L., Hawkins P. (2012). PI3K signalling: The path to discovery and understanding. Nat. Rev. Mol. Cell Biol..

[38] Cantley L.C. (2002). The phosphoinositide 3-kinase pathway. Science.

[39] Datta S.R., Brunet A., Greenberg M.E. (1999). Cellular survival: A play in three Akts. Genes Dev..

[40] Preeti V., Divya V. (2017). Phosphoinositide-3-kinases as the Novel Therapeutic Targets for the Inflammatory Diseases: Current and Future Perspectives. Curr. Drug Targets.

[41] Hawkins P.T., Stephens L.R. (2015). PI3K signalling in inflammation. Biochim. Biophys. Acta (BBA)-Mol. Cell Biol. Lipids.

[42] Li Z., Yang H., Hai Y., Cheng Y. (2023). Regulatory Effect of Inflammatory Mediators in Intervertebral Disc Degeneration. Mediat. Inflamm..

[43] Que Y.C., Wong C.P., Qiu J.C., Gao W.J., Lin Y.X., Zhou H., Gao B., Li P.F., Deng Z.H., Shi H.H. (2024). Maslinic acid alleviates intervertebral disc degeneration by inhibiting the PI3K/AKT and NF-κB signaling pathways. Acta Biochim. Biophys. Sin..

[44] Gong Y.H., Qiu J.X., Jiang T., Li Z., Zhang W.K., Zheng X.H., He Z.X., Chen W.F., Wang Z.F., Feng X.B. (2023). Maltol ameliorates intervertebral disc degeneration through inhibiting PI3K/AKT/NF-κB pathway and regulating NLRP3 inflammasome-mediated pyroptosis. Inflammopharmacology.

[45] Song W., Dai B., Dai Y. (2022). Influence of Ginsenoside Rh2 on Cardiomyocyte Pyroptosis in Rats with Acute Myocardial Infarction. Evid. Based Complement. Altern. Med..

[46] Jin Z.H., Qiu W., Liu H., Jiang X.H., Wang L. (2018). Enhancement of oral bioavailability and immune response of Ginsenoside Rh2 by co-administration with piperine. Chin. J. Nat. Med..

[47] An H., Andersson G., Thonar E., Okuma M., Imai Y., Muehleman C., Aota Y., Masuda K. (2005). A Novel Rabbit Model of Mild, Reproducible Disc Degeneration by an Anulus Needle Puncture: Correlation Between the Degree of Disc Injury and Radiological and Histological Appearances of Disc Degeneration. Spine.

